# *Lactobacillus paracasei* PS23 decelerated age-related muscle loss by ensuring mitochondrial function in SAMP8 mice

**DOI:** 10.18632/aging.101782

**Published:** 2019-01-29

**Authors:** Li-Han Chen, Shih-Yi Huang, Kuo-Chin Huang, Chih-Chieh Hsu, Kuen-Cheh Yang, Lin-Ai Li, Ching-Hung Chan, Hui-Yu Huang

**Affiliations:** ^1^Department of Food Science, Nutrition, and Nutraceutical Biotechnology, Shih Chien University, Taipei 10462, Taiwan; ^2^Graduate Institute of Metabolism and Obesity Sciences, Taipei Medical University, Taipei 11031, Taiwan; ^3^Department of Family Medicine, School of Medicine, College of Medicine, National Taiwan University, Taipei 10002, Taiwan; ^4^Department of Family Medicine, National Taiwan University Hospital, Taipei 10002, Taiwan; ^5^Department of Family Medicine, National Taiwan University Hospital Beihu Branch, Taipei 10845, Taiwan; ^6^Research and Development Department, Bened Biomedical Co., Ltd., Taipei 10448, Taiwan; ^7^Health Science and Wellness Center, National Taiwan University, Taipei 10617, Taiwan; ^*^Equal contribution

**Keywords:** sarcopenia, mitochondrial function, *Lactobacillus paracasei* PS23, age-related inflammation, protein uptake

## Abstract

Sarcopenia is a common impairment in the elderly population responsible for poor outcomes later in life; it can be caused by age-related alternations. Only a few strategies have been reported to reduce sarcopenia.* Lactobacillus paracasei* PS23 (LPPS23) has been reported to delay some age-related disorders. Therefore, here we investigated whether LPPS23 decelerates age-related muscle loss and its underlying mechanism. Female senescence-accelerated mouse prone-8 (SAMP8) mice were divided into three groups (n=6 each): non-aging (16-week-old), control (28-week-old), and PS23 (28-week-old) groups. The control and PS23 groups were given saline and LPPS23, respectively. We evaluated the effects of LPPS23 by analyzing body weight and composition, muscle strength, protein uptake, mitochondrial function, reactive oxygen species (ROS), antioxidant enzymes, and inflammation-related cytokines. LPPS23 significantly attenuated age-related decreases of muscle mass and strength. Compared to the control group, the non-aging and PS23 groups exhibited higher mitochondrial function, IL10, antioxidant enzymes, and protein uptake. Moreover, inflammatory cytokines and ROS were lower in the non-aging and PS23 groups than the control group. Taken together, LPPS23 extenuated sarcopenia progression during aging; this effect might have been enacted by preserving the mitochondrial function via reducing age-related inflammation and ROS and by retaining protein uptake in the SAMP8 mice.

## INTRODUCTION

Aging-associated diseases represent a growing issue in modern society due to an ever-increasing proportion of elderly individuals. Sarcopenia is one of the common geriatric syndromes; its prevalence is estimated to be up to 35% in hospital wards [1, 2]. Sarcopenia is the progressive loss of skeletal muscle mass and strength, and it results in negative health outcomes in late life [3]. Therefore, investigating sarcopenia is an important task of ‘healthy aging.’

Mitochondrial dysfunction is one of the major factors contributing to age-related sarcopenia [4, 5]. Because the vital functions of mitochondria are energy provision, redox homeostasis, and regulation of several catabolic pathways, mitochondrial function is linked to maintenance of muscle [5]. In skeletal muscle, age-related mitochondrial dysfunctions include declines in O_2_ consumption [6, 7], mitochondrial biogenesis [8], mitochondrial mass [9], activities of tricarboxylic acid cycle enzymes [10], and ATP synthesis [11]. Coen et al. (2013) reported that the age-related decline of mitochondrial ATP synthesis/O_2_ consumption was perfectly correlated with walking speed in the elderly [7]. Bogengler *et al.* (2017) also suggested that improving mitochondrial function could attenuate the age-associated rate of muscle loss and functional decline in their review [5]. Therefore, preventing age-related mitochondrial dysfunction should attenuate the incidence of sarcopenia.

Age-related inflammation is involved in age-related sarcopenia through mitochondrial dysfunction [12]. Picca *et al.* (2018) reported a mechanism of sarcopenia development in which age-related inflammation induced mitochondrial dysfunction followed by increases of reactive oxygen species (ROS) and pro-inflammatory cytokines, and subsequently led to further mitochondrial damage, ultimately establishing a cycle contributing to sarcopenia [12]. Because age-related inflammation is linked to a reduction of interleukin (IL)-10 [13, 14], preserving the level of anti-inflammatory cytokine IL10 during aging should disrupt the cycle of inflammation and mitochondrial dysfunction, thereby improving sarcopenia.

IL10 suppresses pro-inflammatory cytokines and chemokines, such as IL6, tumor necrosis factor α (TNFα), and Monocyte chemoattractant protein-1 (MCP1) [15–18]. Although IL6 can activate the anti-inflammation and muscle proliferation responses after acute exercise [19], it is usually considered as a pro-inflammatory cytokine involved in the development of age-related inflammation and sarcopenia [20–22]. Because increases of IL6, TNFα, and MCP1 were reported as biomarkers and reasons for age-related inflammation and sarcopenia [17, 18, 23, 24], IL10 could reduce these pro-inflammatory cytokines and mitigate age-related inflammation and sarcopenia.

Mitochondrial function is also influenced by ROS. The mitochondrial free radical theory of aging indicates that oxidative damage to mitochondrial DNA (mtDNA) causes oxidative phosphorylation impairment and a decrease in ATP production and ROS generation [25]. The observation that the mice expressing an error-prone mtDNA polymerase-γ developed sarcopenia at a young age also provides evidence linking mtDNA damage, mitochondrial dysfunction, and muscle atrophy [26–28]. Thus, decreasing ROS could modulate mitochondrial dysfunction and result in mitigation of age-related sarcopenia.

Probiotics have been reported to have anti-inflammation, anti-ROS, and regulating mitochondrial function properties that are associated with the prevention of sarcopenia [29–31]. Also, probiotics could modulate gut microbiota, which is related to sarcopenia [32, 33]. Moreover, the previous studies revealed that the gut microbiota could enhance protein absorption [34–36]. Due to a positive effect of protein uptake on the prevention of sarcopenia [33], probiotics might also be able to reduce sarcopenia. Although sarcopenia is extenuated by food supplements that have anti-ROS capabilities [37–39], probiotics should provide a more comprehensive effect on sarcopenia because probiotics have several other capabilities that are linked to improvement of sarcopenia. However, there have been no studies investigating the effect of probiotics in age-related sarcopenia.

Elderly mice would be the most physiological model for aging-related studies, but it is expensive and requires much time because the breeding period of experimental animals is relatively long. Therefore, senescence-accelerated animal models are often used in age-related research. Senescence-accelerated mouse prone 8 (SAMP8) was developed by Takeda *et al.* (1981) and characterized by an early onset of age-related alterations [30]. Moreover, several previous studies have used the SAMP8 mouse as an animal model of aging-related muscle and mitochondrial dysfunction [40–44]. According to a previous study, SAMP8 mice are characterized by an early onset of age-related alterations and their aging begins rapidly after 4 months of age [45]. Therefore, in the present study,* Lactobacillus paracasei* PS23 (LPPS23) was given to SAMP8 mice ranging in age from 16 weeks to 28 weeks.

To investigate the effect of probiotics on preventing sarcopenia, we used LPPS23 to treat SAMP8 mice from their youth into old age. The effect of LPPS23 and its underlying mechanism were studied by determining muscle mass, muscle strength, mitochondrial function, inflammation-related cytokines and chemokine, anti-ROS capability, and protein digestibility.

## RESULTS

### Effects of LPPS23 on body weight, body composition, fecal protein, and protein digestibility

**Figure 1:**
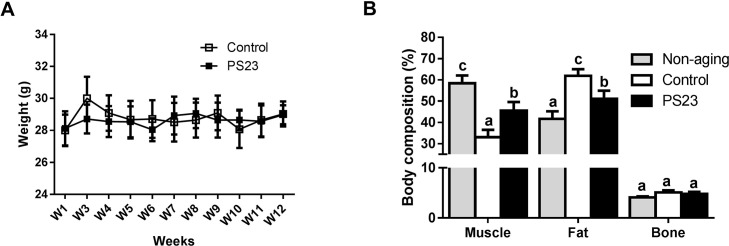
**Body weight and body composition.** (**A**) Average weight was measured from weeks 1–12. (**B**) Body composition was detected at the 12^th^ week. Different superscript letters (a, b, c) differ significantly at *p* < 0.05 according to one-way ANOVA with a Tukey HSD post-hoc test.

The SAMP8 mice were fed identical diets, and each mouse’s average food intake was between 3.5 g/day and 4.5 g/day for the 12 weeks. Although body weight did not differ between the control and PS23 groups in the 12 weeks (Figure 1A), mice in the PS23 group exhibited a higher percentage of muscle and a lower percentage of fat in the end. The proportion of muscle and fat were the highest and lowest, respectively, in the non-aging group. There was no difference among groups in terms of the percentage of bone (Figure 1B). Total food and protein intake were similar among the control and PS23 groups (Table 1), and the level of fecal protein was higher in the control than PS23 groups. We also calculated protein digestibility [(total protein intake – total protein in stool)/total protein intake] to assess the protein uptake. The protein digestibility was higher in the PS23 group than in the control group (Table 1).

**Table 1:** Body weight gain, total food intake, fecal protein, and protein digestibility in aged SAMP8 mice

	Control	PS23
Body weight gain (g) /mouse	0.3±0.2	0.4±1.2
Total food intake (g) /mouse	292.1±4.2	288.8±17.1
Average protein intake (g) /mouse/day	0.87±0.01	0.86±0.05
Fecal protein (g)/mouse/day^#^	0.15±0.01	0.08±0.01^*^
Protein digestibility (%)	81.23±1.7	89.81±0.91^*^

All values are means ± SEM (n = 6 mice/group). ^#^Determined in 3 days for calculating protein digestibility. ^*^Significantly different from the control group at *p* < 0.05 according to a two-tailed Student’s *t*-test.

### Holding impulse and grip force

**Figure 2:**
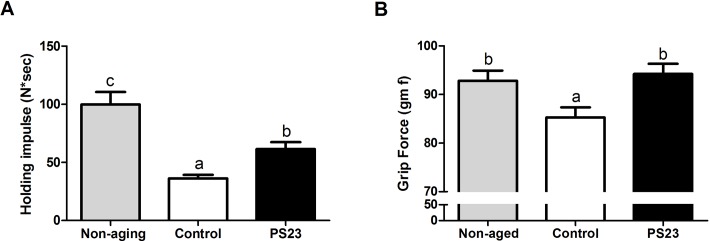
**Muscle strength.** (**A**) Holding impulse and (**B**) group force evaluated the muscle strength of SAMP8 mice. Different superscript letters (a, b, c) differ significantly at *p* < 0.05 according to one-way ANOVA with a Tukey HSD post-hoc test.

Because sarcopenia was defined as a progressive loss of skeletal muscle mass and strength, we then evaluated the effects of LPPS23 on the other feature of sarcopenia, muscle strength. The muscle strength was assessed utilizing the four-limb hanging test and the grip strength test. The holding impulse (Figure 2A) and grip force (Figure 2B) were reduced by 64% and 8%, respectively, in the aged mice (control) compared with non-aging mice. LPPS23 significantly attenuated the age-related reduction of the holding impulse (Figure 2A) and grip force (Figure 2B). The results indicated that LPPS23 can maintain muscle strength during aging in mice.

### Oxygen consumption rate (OCR) of muscle

**Figure 3:**
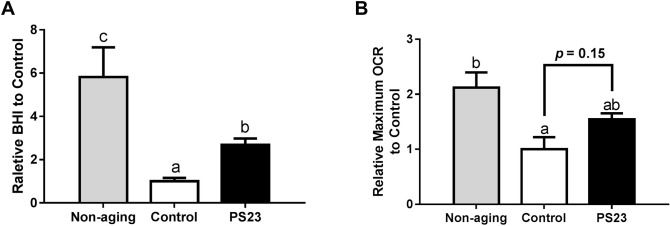
**The oxygen consumption rate of muscle in SAMP8 mice.** (**A**) Relative BHI; (**B**) Relative maximum OCR. Different superscript letters (a, b, c) differ significantly at *p* < 0.05 according to one-way ANOVA with a Tukey HSD post-hoc test.

Muscle weakness can be caused by a decline in mitochondrial function [46]. Therefore, we assessed mitochondrial function by determining the OCR of muscle using an XF24 Extracellular Flux Analyzer (Seahorse Bioscience). The non-aging group exhibited the highest Bioenergetic Health Index (BHI) and maximum OCR among the three groups. The BHI of the PS23 group was 2.7 times that of the control group. Moreover, the PS23 group exhibited a 1.5-times-higher maximum OCR than the control group. However, this difference was not statistically significant (*p* = 0.15) (Figure 3A and 3B). According to the results of BHI and maximum OCR, LPPS23 extenuates the speed of age-related mitochondrial impairment.

### The expressions of genes related to mitochondrial biogenesis in muscle

**Figure 4:**
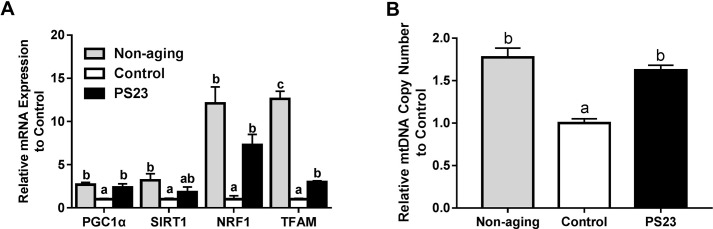
**Mitochondrial biogenesis and mtDNA copy number.** (**A**) Relative mRNA expressions of genes related to mitochondrial biogenesis; (**B**) Relative mtDNA copy number. Different superscript letters (a, b, c) differ significantly at p < 0.05 according to one-way ANOVA with a Tukey HSD post-hoc test.

Dysregulation of mitochondrial biogenesis is typically associated with aging and results in mitochondria dysfunction [6, 8]. Therefore, we measured the genes involved in mitochondrial biogenesis including *PGC1α*, *SIRT1*, *NRF1*, and* TFAM*. The lower levels of the genes in the control group compared with the non-aging groups indicated that mitochondrial biogenesis decreased with age in SAMP8 mice. Although the levels of *NRF1* and *TFAM* were also lower in the PS23 group than in the non-aging group, mice in the PS23 group exhibited higher levels of *PGC1α*, *NRF1*, and* TFAM* compared with mice in the control group (Figure 4A). Our results suggest that LPPS23 can prevent a decline in mitochondrial biogenesis.

### mtDNA copy number

Next, we studied the effect of LPPS23 on mtDNA copy number because the decline of mtDNA abundance was suggested to drive the aging process in skeletal muscle [47]. The results showed that mtDNA copy number decreased in the control group compared to the non-aging group. On the other hand, higher mtDNA copy number was observed in the PS23 than control groups, and no difference was between the PS23 and non-aging groups (Figure 4B).

### Pro-inflammatory cytokines and chemokine in serum

**Figure 5:**
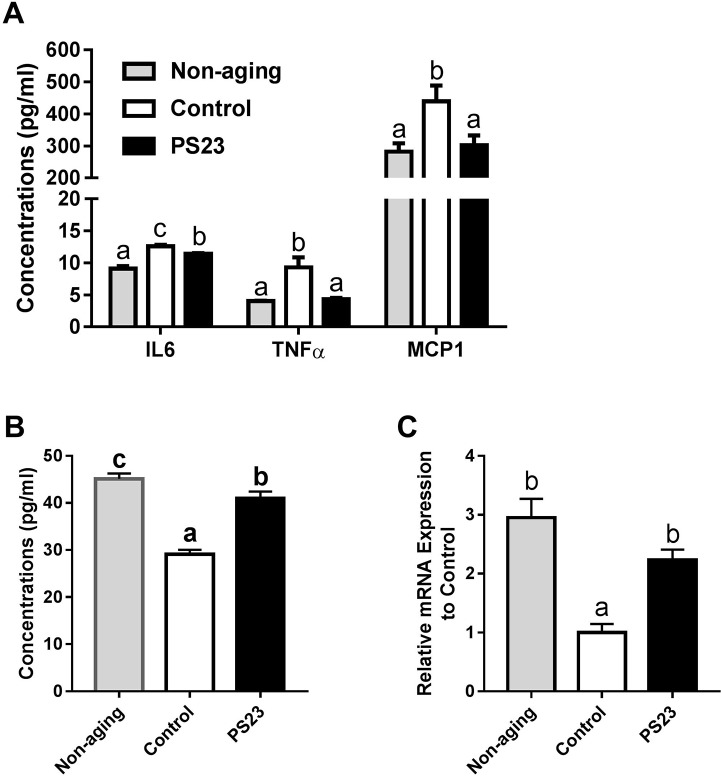
**Levels of inflammation-related cytokines in serum and muscle.** (**A**) The serum levels of IL6, TNFα, and MCP1; (**B**) The serum level of IL10; (**C**) The mRNA level of IL10 in muscle. Different superscript letters (a, b, c) differ significantly at p < 0.05 according to one-way ANOVA with a Tukey HSD post-hoc test.

An age-related inflammation has been reported to correlate with age-related muscle weakness [48]. Compared with the non-aging group, the control group had 38.5% more IL-6 in their serum, and the PS23 group had 25.3% more IL6 in their serum. LPPS23 decreased 10% of IL6 in the serum of aged mice. Moreover, the expression of TNFα was the lowest in the non-aging group followed by the PS23 and control groups, respectively. Similarly, MCP1 is lower in the PS23 and non-aging than control groups (Figure 5A). Thus, LPPS23 reduced the age-related inflammation.

### Levels of IL-10 in serum and muscle

We also measured the protein and mRNA levels of IL10, an anti-inflammatory cytokine, in serum and muscle, respectively. The levels of IL10 were the lowest in the control group in both serum and muscle samples. Compared to the non-aging groups, the PS23 group showed significantly lower serum level of IL10 and no statistical different mRNA level of IL10 in muscle (Figure 5B and 5C). Therefore, LPPS23 can decrease age-related alternations of IL10 level.

### Oxidative stress and antioxidant enzymes in muscle

**Figure 6:**
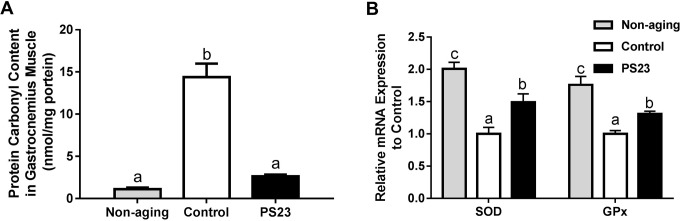
**Protein carbonyl content (A) and mRNA expressions of anti-oxidant enzymes (B) in muscle.** Different superscript letters (a, b, c) differ significantly at p < 0.05 according to one-way ANOVA with a Tukey HSD post-hoc test.

Mitochondrial function is also influenced by oxidative stress that increases with aging. The levels of protein carbonyl groups are an important and immediate biomarker of oxidative stress [49]. The protein carbonyl content in the control group was the highest among the groups, and there was only a slightly higher level of protein carbonyl groups in the PS23 than non-aging groups, but no statistical difference (Figure 6A). Therefore, the control mice exhibited more severe oxidative stress than the other groups. For investigating the possible mechanism, we further detected the mRNA levels of antioxidant enzymes, superoxide dismutase (SOD) and glutathione peroxidase (GPx). The levels of SOD and GPx were enhanced in the PS23 group compared to the control group (Figure 6B). The results demonstrated that LPPS23 modulated the age-related decline of ROS-scavenging capability.

## DISCUSSION

Sarcopenia refers to the age-related loss of skeletal muscle mass and function and is accompanied by physical frailty and an increased risk of morbidity [50]. In the present study, we provided the first evidence that LPPS23 can attenuate sarcopenia progression in SAMP8 mice. LPPS23 administration can impede muscle mass loss and decrease muscle weakness to delay sarcopenia progression. Moreover, LPPS23 was able to maintain mitochondrial function, preventing age-related inflammation, balancing ROS, and enhancing the protein digestibility. Our results suggest that oral administration of LPPS23 might delay the age-related development of sarcopenia by ensuring mitochondrial function through disrupting the sarcopenia-inducing cycle of inflammation, mitochondrial dysfunction, and ROS hyper-production in SAMP8 mice.

Aging-related sarcopenia is a serious problem with few effective therapies. Although a few previous studies have suggested that balancing the composition of gut microbiota and supplementing with prebiotics might improve muscle mass and function in the elderly [32, 33, 51], the effect of probiotics on age-related sarcopenia remains unknown. To the best of our knowledge, only one study regarding the effect of probiotics on muscle revealed that the treatment of *Lctobacillius* *plantarum* TWK10 increased the muscle mass of young-adult ICR mice [52]. Herein, we demonstrate that LPPS23 supplementation decelerated age-related declines of muscle proportion and strength in SAMP8 mice. Therefore, probiotics also have a positive effect on age-related sarcopenia in mice and could be a potential therapeutic agent of sarcopenia.

An increasing number of studies are indicating that gut microbiota plays an important role in maintaining the host’s functions, such as its metabolism, cognition, and immunity [53–55]. Gut microbiota might be involved in the onset and clinical course of sarcopenia [56, 57]. The gut-muscle axis is a new concept based on the correlation between gut microbiota composition and muscle function. According to this concept, some key microbial taxa influence muscle structure and function from the gut via regulating inflammation, ROS, and mitochondrial function in skeletal muscle [33]. Supplementing probiotics possibly modulates sarcopenia by adjusting the composition of gut microbiota and the microbiota subsequently metabolizing some nutrients, including fibers and proteins, into mediators, such as short-chain fatty acids, which could enter the systemic circulation. These mediators have a known influence on myocytes and their mitochondria via multiple signaling pathways that result from the modulation of inflammation and the promotion of insulin sensitivity [58]. Although the effect of LPPS23 on microbiota has not been revealed, several studies have shown that supplementing probiotics at 10^9^ CFU/day/mouse changes the composition of gut microbiota [59–61]. Therefore, LPPS23 might employ the gut-muscle axis to prevent sarcopenia. However, further study is needed to reveal the effect of LPPS23 on gut microbiota.

Mitochondrial dysfunction reduces ATP production. ATP reduction could be the basis of reduced muscle protein turnover. Moreover, dysfunctional mitochondria increases inflammation and ROS that are strongly linked to sarcopenia [12]. Therefore, ensuring mitochondrial function is an important strategy for preventing muscle loss [5]. In our study, LPPS23 lowered the aging-related mitochondrial alternations by regulation of *PGC1α*,* SIRT1*,* NRF1*, and* TFAM* gene expression. The levels of OCR and genes involved in mitochondrial biogenesis (e.g., *PGC1α*, *SIRT1*,* NRF1*, and *TFAM*) were reduced in the control group, which demonstrated that the impairment of mitochondrial dysfunction occurred during aging in the SAMP8 mice. Moreover, the slighter decline of mtDNA copy number in the PS23 than control groups indicates that LPPS23 reduces mitochondrial damage during aging.

How does LPPS23 protect mitochondrial function to prevent sarcopenia? One possible mechanism is that LPPS23 attenuates the age-related inflammation. Age-related inflammation is not only involved in the gut-muscle axis but is also a major aetiological factor in the development of sarcopenia [12, 32, 36, 58, 62]. Age-related inflammation can initiate a cycle of inflammation, mitochondrial dysfunction, and ROS hyper-production, and this cycle contributes to sarcopenia [12]. Therefore, to extenuate age-related inflammation, efforts should be aimed at disrupting this cycle and improving sarcopenia. IL10 is an anti-inflammatory cytokine, and the decline of IL10 is suggested to cause aging-associated inflammation [13, 14]. Therefore, enhancing the level of IL10 should reduce age-related inflammation. Several Lactobacillus spp. were reported for their IL10-mediated effect on anti-inflammation. For example, *Lactobacillus paracasei* ST11 showed anti-inflammation capability associated with strong induction of IL10 [29]. *Lactobacillus casei* strain Shirota was reported to improve chronic inflammation by increasing the level of IL10 [30]. *Lactobacillus salivarius* Ls33 rescued mice from colitis in an IL10-dependent manner. *Lactobacillus paracasei* NCC2461 modulates allergic airway inflammation [63]. Therefore, it was not surprising to observe that LPPS23 increased IL10 and decreased TNFα, IL6, and MCP1 in elderly SAMP8 mice in our study. Taken together, our data show that LPPS23 might reduce age-related inflammation by preserving the level of IL10 in aged SAMP8 mice, thereby preventing mitochondrial dysfunction and ROS production and ultimately improving sarcopenia.

ROS is another dominant factor leading to mitochondrial dysfunction and sarcopenia [64]; reducing ROS has been shown to improve the sarcopenia resulting from mitochondrial dysfunction [46]. In the present study, we observed lower ROS levels and mitochondrial dysfunction in the non-aging group compared to the control group. Therefore, age-related sarcopenia in SAMP8 could be caused by an increase in ROS during aging. We also demonstrated that LPPS23 has an anti-ROS capability because the PS23 group had lower ROS and higher expressions of anti-oxidant enzymes (SOD and GPx) in the muscle than the control group. These results indicate a possible anti-sarcopenia mechanism of LPPS23 that increases the anti-oxidant enzymes to interrupt the cycle of inflammation, mitochondrial dysfunction, and ROS hyper-production. Similar to LPPS23, some probiotics have been reported to have anti-ROS properties [31, 65], so other probiotics should be tested for their usefulness for modulating sarcopenia.

A high ratio of muscle protein breakdown (MPB) to muscle protein synthesis (MPS) is another possible reason for sarcopenia. Thus, a reduction in MPB and/or a rise in MPS were suggested to result in the accretion of muscle [66]. Inflammation was linked to the activation of catabolism and the down-regulation of anabolism in the muscle protein [67]. Moreover, some studies have shown that protein supplementation can positively correlate with protein synthesis in older populations [68–71]. Because gut microbiota can influence protein uptake [34–36], it is not surprising that Jager et al. reported that probiotics, including Bacillus coagulants GBI-30, could improve protein uptake and utilization [72]. In the current study, protein intake was not different between the control and PS23 groups, but the level of fecal protein was significantly lower in the PS23 than control groups. We further revealed that the PS23 group had higher protein digestibility than the control group. The similar levels of protein intake and higher levels of protein digestibility in the PS23 than control groups indicates that LPPS23 assisted aged mice to retain more protein and might help aged mice to increase their protein uptake for balancing MPB and MPS. Since LPPS23 decreased the age-related inflammation and seemed to increase the protein uptake in the aged SAMP8, LPPS23 could also be assumed to improve sarcopenia by balancing the MPB and MPS. However, the age-related alternation of MPB and MPS remains controversial. Therefore, more studies are needed to demonstrate whether LPPS23 regulates MPB and MPS to reduce sarcopenia.

Based on our findings, LPPS23 can affect mitochondrial function and oxidative stress in the aged mice. This raises the question of whether these effects of LPPS23 also appear in the non-aging mice. Since LPPS23 seems to influence age-related effects of mitochondrial function and oxidative stress by reducing the age-related inflammation, the effects of LPPS23 on mitochondrial function and oxidative in health non-aging mice might not be as significant as those reported in the present study. However, the anti-inflammatory and anti-ROS capabilities of LPPS23 were shown in the aged mice. Moreover, *Lactobacillus* spp. were reported for their capabilities of anti-ROS and mitochondrial regulation [65, 73, 74]. Therefore, we cannot exclude the possibility that LPPS23 influences mitochondrial function and oxidative stress in non-aging mice. It will be interesting to understand whether LPPS23 affects mitochondrial function and oxidative stress in non-aging mice.

The dose of LPPS23 used in the present study was obtained from previous studies [59–61, 75] and our pilot study. In the pilot study, the mice were given LPPS23 at either 1 × 10^8^ or 1 × 10^9^ CFU/mouse /day. Although giving mice LPPS23 at 1 × 10^8^ CFU/mouse /day also improved the muscle mass and muscle strength, there were no significant differences between the mice given saline and LPPS23 at 1 × 10^8^ CFU/mouse/day. Because the major purpose of the study was to demonstrate the effect of LPPS23 on the improvement of sarcopenia, we only treated mice with LPPS23 at 1 × 10^9^ CFU/mouse /day in the formal study to reduce the cost of animal life and budget. However, this dose-dependent response might assist us to understand the effect of LPPS23 on sarcopenia more comprehensively and would be an interesting topic of further study.

In conclusion, this study is the first to report the preventative effect of probiotics on sarcopenia in SAMP8 mice. Our results also suggest that LPPS23 employs the anti-inflammation and anti-ROS capabilities to maintain mitochondrial function, ultimately hindering the progression of sarcopenia. Moreover, the enhancement the protein uptake might be another mechanism used against sarcopenia by LPPS23. Therefore, LPPS23 supplementation could be a potential strategy for delaying the progression of the age-related disease known as sarcopenia.

## MATERIALS AND METHODS

### *L. paracasei* PS23 and experimental animals

The LPPS23 was provided by Dr. Ying-Chieh Tsai (National Yang-Ming University, Taipei, Taiwan). The concentration of LPPS23 was adjusted to 1 × 10^9^ CFU/200 μL for subsequent utilization. SAMP8 mice were provided by Dr. Ming-Fu Wang (Providence University, Taichung, Taiwan) and housed in standard laboratory conditions (12/12-h light/dark cycle, 22–24°C, 40–60% humidity). The animals were fed a commercially available diet (local supplier), the ingredients of which are listed in Supplementary Table 1. Sterile water was provided ad libitum. Eighteen female mice were divided into three groups each (n = 6), namely non-aging (sacrificed at week 0), control (fed for 12 weeks and treated with saline until an age of 28 weeks), and PS23 (fed for 12 weeks and treated with LPPS23 [1 × 10^9^ CFU/mouse/day] until an age of 28 weeks) groups. The dose of LPPS23 was chosen based on our previous studies [75]. To ensure that the mice received live LPPS23, the bacteria were delivered by gavage performed by well-trained investigators. The gavages were performed at 9:00 a.m. daily for 12 weeks. Three days after the evaluation of muscle strength utilizing the four-limb hanging test and a grip strength test, we measured the body composition of the control and PS23 groups using Dual-energy X-ray Absorptiometry (DEXA). The animals were then sacrificed at the end of 28 weeks. All of the animal experiments were performed in accordance with protocols approved by the Institutional Animal Care and Use Committee (IACUC) of Shih Chien University (IACUC-10509).

### Protein digestibility

The protein digestibility rate was calculated by [(total protein intake – total protein in stool)/total protein intake]. The stool was collected and weighed for 3 days. The concentration of protein in stool was determined using the BCA Protein Assay Kit (Thermo Fisher Scientific, Waltham, MA, USA) following the manufacturer’s protocols.

### Dual-energy X-ray absorptiometry (DEXA)

DEXA scans were collected on a GE Lumar PIXImus2 (GE, Madison, WI, USA) three days prior to sacrifice. The data were analyzed using software included by the manufacturer.

### Muscle strength testing

The four-limb hanging test was performed three days before sacrifice. The testing procedure was modified based on a protocol previously described [76]. The mice were made to hold on the top of a standard wire cage lid for a few seconds before turning the lid upside down. The holding time was measured. The experiment was repeated three times, and the average value was computed. The results were reported as holding impulse (weight (g) × duration (s) of holding).

A grip strength test was performed three days before sacrifice following the description in a previous study [77]. Briefly, the mice were made to hold onto a horizontal rod using their forelimbs; they were prohibited from using their hindlimbs by a tail restraint. The maximum grip force in 10 trials was recorded. The grip strength was displayed as the grip force (gm f).

### Measurement of oxygen consumption rates

Oxygen consumption rates (OCRs) were determined utilizing an XF24 Extracellular Flux Analyzer (Seahorse Bioscience, North Billerica, MA, USA) following the manufacturer’s protocols. Briefly, gastrocnemius was homogenized using a Polytron homogenizer in ice-cold MAS buffer (115 mM KCl, 10 mM KH_2_PO_4_, 2 mM MgCl_2_, 3 mM HEPES, and 1 mM EGTA) containing 0.2% fatty acid-free Bovine Serum Albumin. The samples were centrifuged at 700*g* for 10 min to remove nuclei and cell debris and then centrifuged at 8000*g* for 10 min to collect the mitochondria. The mitochondria pellets were re-suspended in Mitochondrial Assay Solution (MAS), and we measured protein concentrations using a BCA assay. Subsequently, the isolated mitochondria were seeded at 50 μl (10 μg of protein) per well in XF24 V7 microplates (Seahorse Bioscience). After being centrifuged at 2200*g* for 20 min at 4°C, 625 μl/well of MAS (containing 5 mM Glutamate, 5 mM malate, and 5 mM succinate) was added to the plate at 37°C. The XF24 plate was transferred to an XF24 Extracellular Flux analyzer at 37°C and equilibrated for 10 min. Four assay cycles (30s mix and 3 min determining period) were used to measure basal respiration. Next, we added oligomycin (4 μM; inhibiting ATP synthase) by automatic pneumatic injection (three assay cycles). Carbonyl cyanide p-trifluoromethoxyphenylhydrazone) (0.5 μM) was then injected to uncouple the mitochondria completely. Finally, a cocktail of ofrotenone (4 μM) and antimycin A (2 μM) was injected to correct for a non-mitochondrial respiratory rate. Oxygen consumption rate measurements were recorded at set interval time points. All materials in the assay were purchased from Sigma-Aldrich (Saint Louis, MO, USA). The BHI was calculated following the description of [78] using the following equation: BHI = log [(reserve capacity × ATP-linked) / (non-mitochondrial × proton leak)].

### RNA extraction and quantitative RT-PCR

RNA was isolated from tissues using an RNeasy Mini kit (Qiagen, Hilden, Germany), and 500 ng of RNA from each sample was reverse-transcribed using an iScript cDNA Synthesis Kit (Bio-Rad, Hercules, CA, USA), following the instructions of the manufacturer. Quantitative PCR was performed in a MyiQ Single-Color Real-Time PCR Detection System (Bio-Rad). The primers of the genes (Purigo, Taipei, Taiwan) are listed in Supplementary Table 2. β-ACTIN was used as an internal control for normalizing the mRNA levels of the genes.

### DNA extraction and quantitative PCR

Total DNA was purified from tissue utilizing DNeasy Blood & Tissue Kits (Qiagen) following the description in manual. q-PCR was performed in a MyiQ Single-Color Real-Time PCR Detection System (Bio-Rad). Mitochondrial quantity was evaluated by normalizing the COXII gene amplification level against the nuclear 18s rRNA gene. The primers of the genes (Purigo) are listed in Supplementary Table 2.

### Protein carbonyl content

The concentration of protein carbonyls was determined spectrophotometrically using a protein carbonyl assay kit (Cayman Chemicals, Ann Arbor, MI, USA) according to the manufacturer’s instructions. Briefly, 200 mg of gastrocnemius was homogenized in phosphate buffer, pH 6.7, and then centrifuged at 10,000*g* for 15 min at 4°C to remove debris. The sample was subsequently incubated with dinitrophenylhydrazine for 1 h at room temperature (RT). The protein was precipitated with trichloroacetic acid and resuspended in guanidine hydrochloride. After being centrifugated at 10,000*g* for 10 min at 4°C, the absorbance of the supernatant was measured at 370 nm.

### Determination of cytokines in plasma

The inflammation-related cytokines were determined from plasma using the Mouse IL-6 ELISA MAX™ Standard, Mouse TNF-alpha ELISA MAX Standard, and Mouse MCP-1 ELISA MAX Standard (Biolegend, San Diego, CA, USA) according to the manufacturer's instructions. In brief, samples and standards were added to a pre-coated 96-well plate and incubated for 2 h. Next, either anti-mouse IL-6, TNFα, or MCP1 antibody was added, and the mixture was incubated for 2 h. Detection antibody was then added to each well, and the mixture was incubated at RT for 1 h. Following incubation with Avidin-HRP solution at RT for 30 minutes, TMB Substrate Solution was added, and the mixture was incubated at RT for 20 min. The reactions were terminated using Stop Solution, and absorbance was read at 450 nm using an ELISA reader (BioTek, Winooski, VT, USA).

### Statistical analyses

The data were presented as means ± SEM. The data were analyzed using one-way ANOVA with a Tukey HSD post-hoc test except for two-tailed Student’s t-test for body weight gain, total food intake, average protein intake, fecal protein, and protein digestibility. A p-value < 0.05 was considered to be statistically significant.

## SUPPLEMENTARY MATERIAL

Supplementary Tables
