# Supplementary material for: Lactobacillus paracasei PS23 decelerated age-related muscle loss by ensuring mitochondrial function in SAMP8 mice
Source: Aging (Albany NY). 2019 Jan 29;11(2):756–70. doi: 10.18632/aging.101782 (PMC6366975; doi:10.18632/aging.101782)
Supplement: Supplementary Tables [file aging-11-101782-s001.pdf]

SUPPLEMENTARY TABLES

Supplementary Table 1. Ingredient composition of diet fed to SAMP8 mice.

Ingredient composition	Normal diet
Corn starch	46.23%
Dextrin	15.38%
Casein-vitamin free	13.89%
Sucrose	9.92%
Fructose	0.00%
Powdered cellulose	4.96%
Soybean Oil	3.97%
AIN 93M Mineral Mix	3.47%
AIN 93 Vitamin Mix	0.99%
Choline Bitartrate	0.23%
L-Cystine	0.17%
t-Butylhydroquinone	0.79%
Kcal/g	1.94

Supplementary Table 2. Primer sequences.

Gene symbols	Accession numbers	Primer sequences
β-ACTIN	NM_031144.3	ACAGGATGCAGAAGGAGATTAC<br>ACAGTGAGGCCAGGATAGA
PGC-1α	NM_133218.2	GTCTCGACACGGAGAGTTAAA G<br>CTAGCCATGGATGGCCTATTT
SIRT1	NM_019812.3	GTTACTGCCACAGGAACTAGAG<br>GTAAGCGGCTTGAGGGTAAT
NRF1	NM_010938.4	GGGAGTCTTCATCAGCACTTAG<br>CTGAACACATGGCTACCATAGA
TFAM	NM_009360.4	TTTTCCAAGCCTCATTTACAAGC<br>AAGACCTCGTTCAGCATATAACATT
COXII	LC061971.1	GCCGACTAAATCAAGCAACA<br>CAATGGGCATAAAGCTATGG
18s rRNA	NR_003278.3	CATTCGAACGTCTGCCCTATCA<br>GGGTCGGGAGTGGGTAATTTG
SOD	NM_013671	GTAGAGCCTTGCCTGTCTTATG<br>AAACCCAGAGGCACCATTAC
GPx	NM_008160.6	CGACATCGAACCTGACATAGAA<br>CAGAGTGCAGCCAGTAATCA
